# Long noncoding RNA lncHERG promotes cell proliferation, migration and invasion in glioblastoma

**DOI:** 10.18632/oncotarget.22446

**Published:** 2017-11-14

**Authors:** Jian Shi, Yong-Jie Wang, Chong-Ran Sun, Bin Qin, Yang Zhang, Gao Chen

**Affiliations:** ^1^ Department of Neurosurgery, The Second Affiliated Hospital of Zhejiang University School of Medicine, Hangzhou 310000, China

**Keywords:** lncHERG, proliferation, migration, miR-940, glioblastoma

## Abstract

Long noncoding RNAs have recently been proven to regulate tumorgenesis in many cancers. However, their biological functions in glioblastoma remain largely unknown. Here we found an uncharacteristic lncRNA lncHERG that is highly expressed in human glioblastoma (GBM). We found that lncHERG knockdown inhibited cell proliferation, migration and invasion in glioblastoma *in vitro* and *in vivo*. Moreover, the higher expression of lncHERG in patients with glioblastoma indicated lower survival rate and poorer prognosis. Mechanistically, we found that lncHERG can serve as a sponge for miR-940 which is a tumor suppressor in cervical cancer and whose function has not been defined in glioblastoma. We showed that miR-940 was down-regulated in glioblastoma tissues compared to peritumor tissues. LncHERG knockdown impaired cell proliferation, migration and invasion while inhibition of miR-940 in the meantime reversed this trend. In conclusion, our study highlights the essential role of lncHERG in glioblastoma by acting as a competing endogenous RNA of miR-940, which may serve as a new prognostic biomarker in glioblastoma.

## INTRODUCTION

Glioblastoma (GBM), the most common and aggressive brain tumor around the world, leads to a large amount of deaths each year [1, 2]. The incidence of glioblastoma is increasing year by year. Surgical resection, radiotherapy and chemotherapy were the main methods for treatment of GBM patients [3–6]. However, the 5-year overall survival rate of patients with GBM is less than 3% [7, 8]. Due to the infiltrative growth of glioblastoma, the rate of tumor recurrence is very high [5]. There is no effective method for fully treatment of glioblastoma so far. Therefore, in order to develop new and effective therapeutic management of GBM, it is an urgent and necessary to define the molecular mechanism that regulates the genesis of glioblastoma.

Long noncoding RNAs (lncRNA) are a class of transcripts whose length is longer than 200 nucleotides and have no protein coding potential [9]. Accumulating evidences show that noncoding RNAs (ncRNA) exert essential roles in all kinds of biological processes including development, immune and especially in tumorgenesis [10–13]. In cancers, lncRNAs have been demonstrated to regulate cell proliferation, migration, invasion and apoptosis by several mechanisms [14]. A lot of lncRNAs are up-regulated or down-regulated in cancers and involved in tumor development and progression [15]. For example, lncRNA AFAP1-AS1 increases tumor growth and metastasis in HCC [16]. LncRNA DUXAP8 promotes cell proliferation and migration in gastric cancer [17]. LncRNA UCA1 promotes gallbladder cancer progression via inhibiting p21 and E-cadherin expression [18]. LncRNA CCEPR is upregulated and associated with poor prognosis in urothelial bladder carcinoma [19]. In glioblastoma, lncRNA HOXA-AS3 increases tumor progression and lncRNA TALNEC2 promotes growth of glioblastoma stem cells [6, 20]. Nevertheless, there are still many uncharacteristic lncRNAs whose functions remain to be illustrated in glioblastoma. Hence, to understand the molecular biology of glioblastoma, it is important to identify more tumor-related lncRNAs.

In our study, we found an uncharacteristic lncRNA lncHERG (Highly expressed long noncoding RNA in glioblastoma) whose expression was higher in glioblastoma than non-tumor tissues. We showed that lncHERG depletion impaired proliferation, migration and invasion of glioblastoma *in vitro* and *in vivo*. What's more, the higher expression of lncHERG in patients with glioblastoma indicated lower survival rate and poorer prognosis. In mechanism, we found that lncHERG sponged miR-940 in glioblastoma. Moreover, miR-940 was down-regulated in glioblastoma tissues compared to peritumor tissues. LncHERG knockdown impaired cell proliferation, migration and invasion while inhibition of miR-940 in the meantime reversed it. In summary, our study highlights the essential role of lncHERG in glioblastoma by acting as a competing endogenous RNA of miR-940, which may serve as a new prognostic biomarker in glioblastoma.

## RESULTS

### LncHERG is highly expressed in glioblastoma

**Figure 1:**
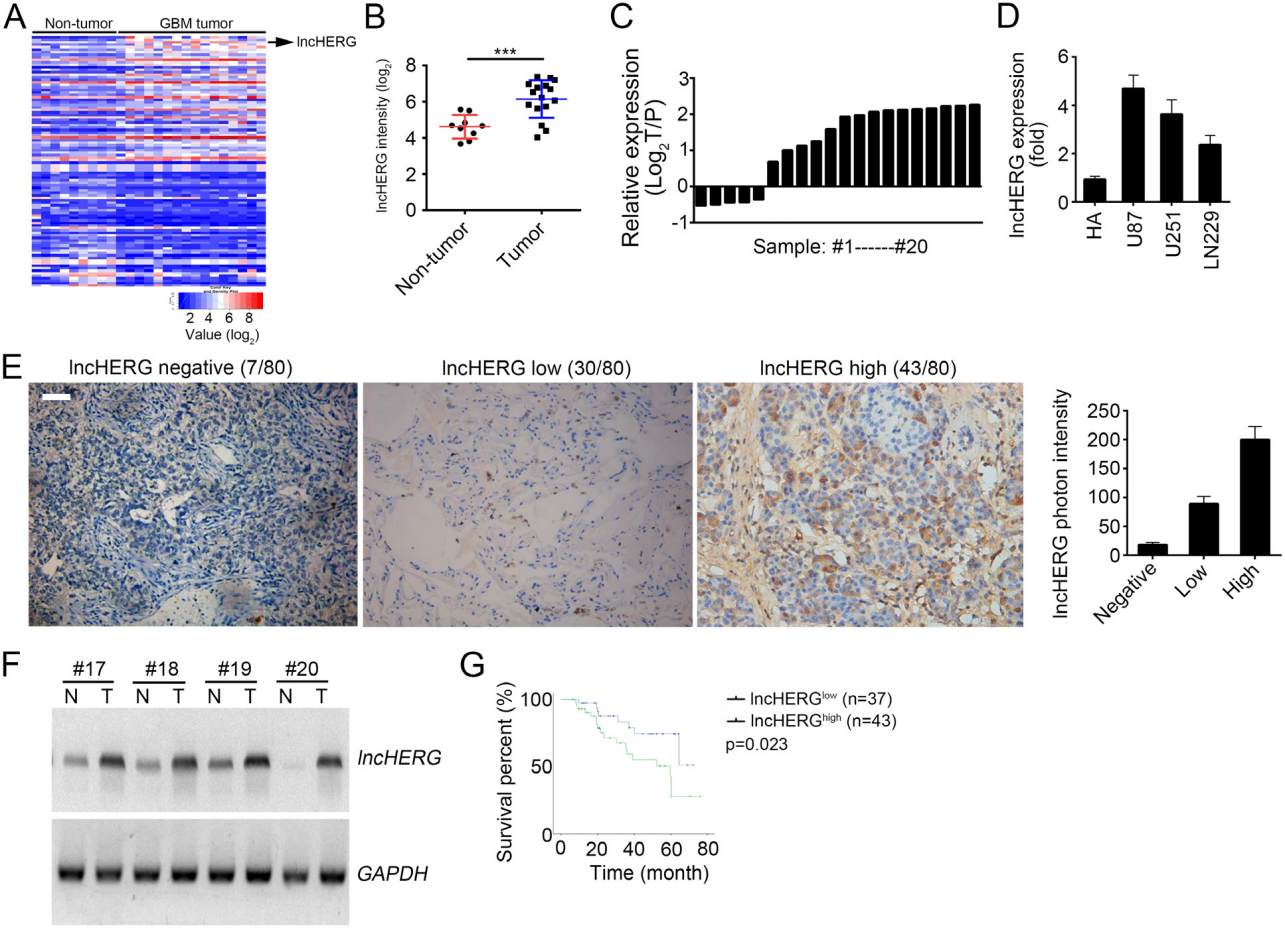
LncHERG is highly expressed in glioblastoma **(A)** LncHERG was up-regulated in glioblastoma (GBM) tissues according to a microarray dataset (GSE90598). **(B)** Analysis of lncHERG expression in non-tumor and GBM tissues according to dataset in GSE90598. **(C)** The expression levels of lncHERG in GBM tissues and paired peritumor tissues were measured. **(D)** The expression of lncHERG in normal glial cell line (HA) and GBM cell lines (U87, U251 and LN229) was analyzed. **(E)** The expression levels of lncHERG in GBM tissues were checked by *in situ* hybridization (ISH). lncHERG was highly expressed in GBM tissues. **(F)** Total RNAs were obtained from GBM tissues and paired peritumor tissues. And lncHERG expression was measured by Northern blot. N, non-tumor; T, Tumor. **(G)** 80 GBM samples were collected and divided into two groups based on lncHERG expression (cut-off: mean value). Then the overall survival rate was analyzed by Kaplan-Meier analysis. All data are representative of three independent experiments and expressed as mean ± SD. ^***^p<0.001.

To explore the biological functions of lncRNAs in glioblastoma, we analyzed an online dataset (GSE90598). We found out an uncharacteristic lncRNA (official symbol: LOC644794) lncHERG that was up-regulated in GBM tissues compared to non-tumor tissues (Figure 1A). lncHERG was significantly up-regulated in glioblastoma (Figure 1B) according to this dataset (GSE90598). To confirm this result, we collected glioblastoma samples and extracted total RNAs. Then we checked the relative expression levels of lncHERG in 20 pairs of glioblastoma samples. We found that lncHERG was also remarkably upregulated in most glioblastoma samples (Figure 1C). Then we checked the expression of lncHERG in normal glial cell line (HA) and GBM cell lines (U87, U251 and LN229). lncHERG was highly expressed in GBM cell lines (Figure 1D). To further validate it, we performed *in situ* hybridization (ISH) assays with specific biotin-labeled probes in 80 glioblastoma samples. We found that most glioblastoma samples showed very high expression of lncHERG (Figure 1E). What's more, we conducted Northern blot assays. As shown, lncHERG was also highly expressed in glioblastoma samples (Figure 1F). Then we divided these samples into two groups according to lncHERG expression levels (mean value was the cut-off). We performed Kaplan-Meier analysis and found that patients with higher lncHERG expression showed lower overall survival rate (Figure 1G). In summary, lncHERG was highly expressed in glioblastoma samples and correlated with tumor prognosis.

### LncHERG promotes cell proliferation and inhibits cell apoptosis

**Figure 2:**
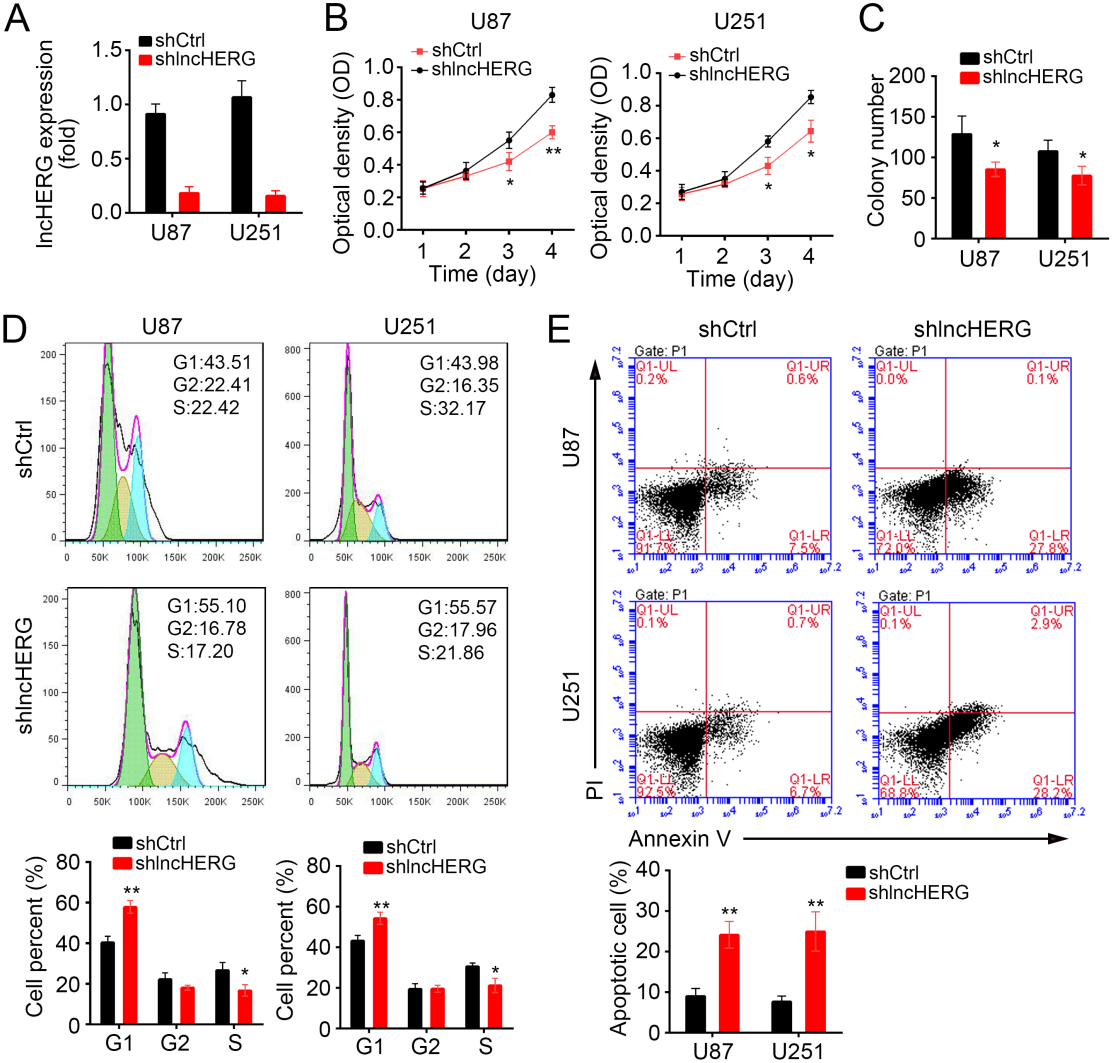
LncHERG promotes cell proliferation and inhibits cell apoptosis **(A)** LncHERG was knocked down by transfection with specific shRNA. The mRNA levels were analyzed by qPCR in lncHERG-depleted cells. **(B)** MTT assays were conducted with shCtrl or shlncHERG U87 and U251 cells. LncHERG knockdown remarkably inhibited cell proliferation. **(C)** LncHERG knockdown impaired the abilities of colony formation of U87 and U251 cells. **(D)** less LncHERG-silenced cells entered into S phase. **(E)** LncHERG depletion dramatically increased the cell apoptosis in U87 and U251 cells. All data are representative of three independent experiments and expressed as mean ± SD. ^*^p<0.05 and ^**^p<0.01.

To define the physiological function of lncHERG in glioblastoma, we knocked down lncHERG in U87 and U251 cells (Figure 2A). Then we performed MTT assays using lncHERG-depleted stable cell lines. We found that lncHERG knockdown significantly inhibited cell proliferation (Figure 2B). What's more, lncHERG-silenced cells formed less clones than shCtrl cells (Figure 2C). Consistently, lncHERG knockdown remarkably impaired cell division. Less shlncHERG cells entered into S phase (Figure 2D). Then we evaluated the cell apoptosis by Annexin V/PI staining. We found that lncHERG silence promoted cell death in U87 and U251 cells (Figure 2E). Collectively, lncHERG enhanced cell proliferation and decreased cell apoptosis in glioblastoma.

### LncHERG enhances cell migration and invasion

**Figure 3:**
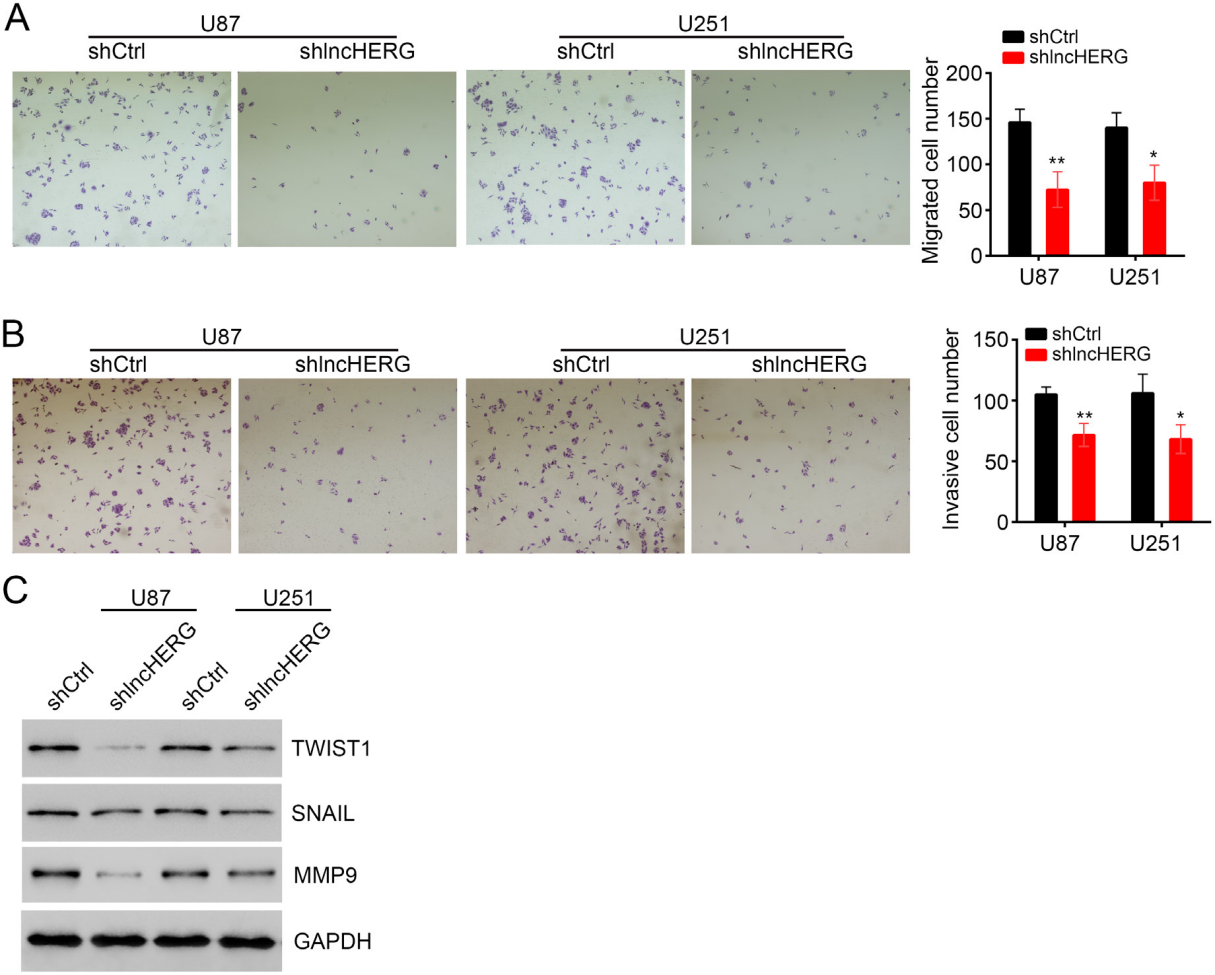
LncHERG enhances cell migration and invasion **(A)** LncHERG knockdown seriously inhibited cell migration. **(B)** LncHERG silence impaired the ability of cell invasion. **(C)** The expression levels of tumor metastasis-related proteins were measured by WB. LncHERG depletion remarkably inhibited cell metastasis in U87 and U251 cells. All data are representative of three independent experiments and expressed as mean ± SD. ^*^p<0.05 and ^**^p<0.01.

Tumor metastasis is the main cause for cancer recurrence and malignance. We wondered whether lncHERG regulated tumor metastasis in glioblastoma. We performed cell migration and invasion assays. After lncHERG knockdown, the abilities of migration and invasion of U87 and U251 cells were dramatically impaired (Figure 3A and 3B). In consistence, the expression levels of metastasis-related proteins (TWIST1, SNAIL and MMP9) were remarkably down-regulated after lncHERG knockdown in U87 and U251 cells (Figure 3C). In summary, lncHERG promoted tumor metastasis in glioblastoma.

### LncHERG depletion impairs tumor propagation *in vivo*

**Figure 4:**
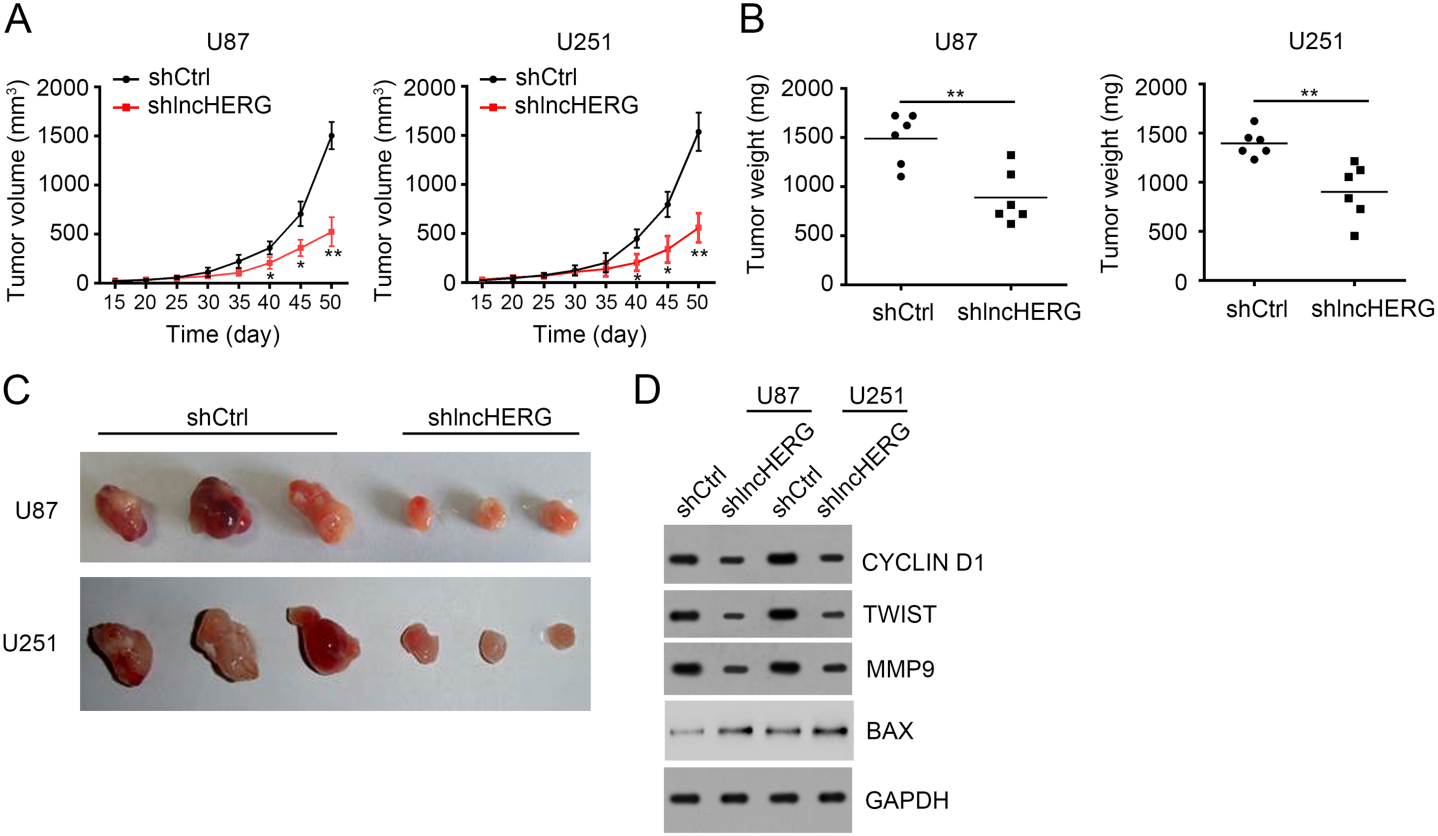
LncHERG depletion impairs tumor propagation *in vivo* **(A)** 4×10^6^ shCtrl or sh lncHERG U87 or U251 cells were subcutaneously injected into 6-week-old Balb/c nude mice. The tumor volumes were measured at different time points. lncHERG knockdown remarkably delayed tumor propagation *in vivo*. **(B)** and **(C)** Tumor weights were analyzed 7 weeks post injection. lncHERG knockdown inhibited tumor growth. **(D)** The proliferation, migration and apoptosis in tumors of C were analyzed by WB. lncHERG depletion seriously inhibited tumor proliferation and migration while promoted apoptosis. All data are representative of three independent experiments and expressed as mean ± SD. ^**^p<0.01.

To further demonstrate the role of lncHERG *in vivo*, we performed tumor xenograft assays. We injected 4×10^6^ shCtrl or shlncHERG U87 or U251 cells into 6-week-old BALB/c nude mice subcutaneously. At indicative time points, we calculated tumor volumes. We found that lncHERG knockdown greatly delayed tumor propagation *in vivo* (Figure 4A). Then 7 weeks later, we measured the tumor weights. We found that tumors derived from shlncHERG U87 or U251 cells were lighter and smaller (Figure 4B and 4C). We then collected the formed tumors and checked cell proliferation, migration, invasion and apoptosis by western blot. We found that tumors originated from shlncHERG U87 or U251 cells showed decreased proliferation, migration and invasion but increased apoptosis (Figure 4D). Summarily, lncHERG regulated cell proliferation, migration, invasion and apoptosis *in vivo*.

### LncHERG sponges miR-940 in glioblastoma

**Figure 5:**
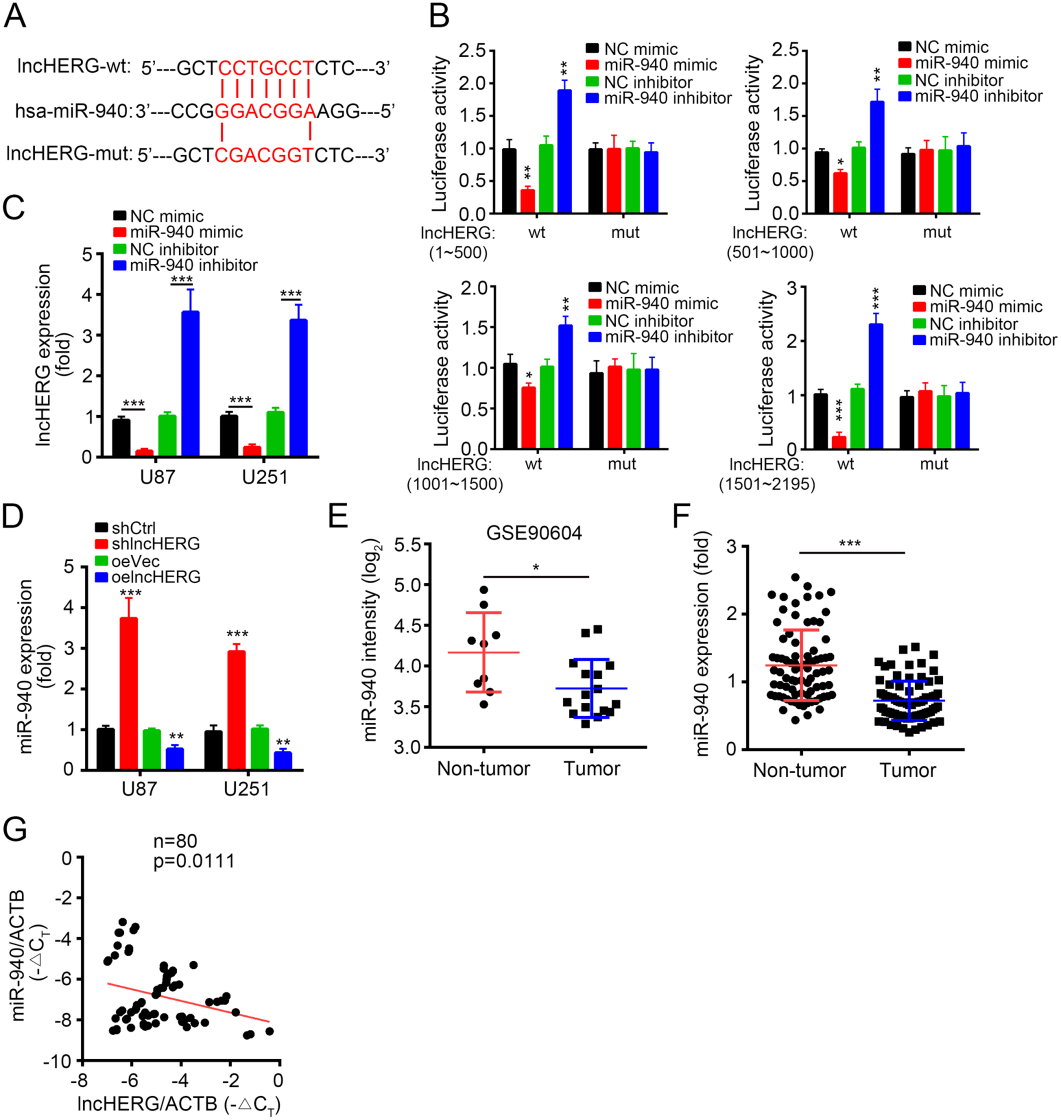
LncHERG sponges miR-940 in glioblastoma **(A)** A diagram for the binding site with miR-940 in lncHERG and construction of lncHERG mutant was shown. **(B)** lncHERG-wt or lncHERG-mut plasmid was transfected into 293T cells with miR-940 mimic or inhibitor for luciferase activity assays. miR-940 mimic remarkably inhibited luciferase activity. **(C)** miR-940 mimic inhibited the mRNA levels of lncHERG in U87 and U251 cells. **(D)** lncHERG overexpression inhibited the expression of miR-940 in U87 and U251 cells. **(E)** miR-940 expression was down-regulated in GBM tissues compared to non-tumor tissues according to a dataset (GSE90604). **(F)** The expression of miR-940 in 80 GBM samples was analyzed. The expression of miR-940 was down-regulated in GBM tissues compared to peritumor tissues. **(G)** The expression of lncHERG was negatively correlated with that of miR-940 in GBM tissues. All data are representative of three independent experiments and expressed as mean ± SD. ^*^p<0.05, ^**^p<0.01 and ^***^p<0.001.

Increasing evidences showed that lncRNAs can serve as competitive endogenous RNAs to bind miRNAs [21]. To search the potential binding miRNAs of lncHERG, we made a prediction by a tool (http://mirdb.org). Some miRNAs were the potential targets of lncHERG, but miR-940 scored top. miR-940 was reported to act as a tumor suppressor. For example, miR-940 inhibited cell proliferation and apoptosis in ovarian cancer by targeting PKC-δ [22]. And miR-940 suppresses cell invasion and migration in HCC by targeting CXCR2 [23]. Besides, miR-940 also inhibited triple-negative breast cancer, pancreatic ductal adenocarcinoma and prostate cancer [24–26]. However, the function of miR-940 in glioblastoma remains elusive. We found that there are 21 potential binding sites with miR-940 in lncHERG (Figure 5A). We divided lncHERG into 4 truncations (1~500bp, 501~1000bp, 1001~1500bp and 1501~2195bp) and constructed four luciferase reporter plasmid and corresponding mutant plasmids (Figure 1A). We then conducted luciferase activity assays by transfecting pRL-lncHERG-wt or pRL-lncHERG-mut together with miR-940 mimic or inhibitor into 293T cells. We found that miR-940 mimic dramatically inhibited the luciferase activity while miR-940 inhibitor promoted the activity (Figure 5B), which demonstrated that lncHERG can bind to miR-940. Then we transfected miR-940 mimic or inhibitor into U87 or U251 cells and checked mRNA levels of lncHERG. We found that miR-940 mimic inhibited lncHERG expression while miR-940 inhibitor enhanced lncHERG expression (Figure 5C). Consistently, lncHERG knockdown promoted the expression of miR-940 while overexpressing lncHERG inhibited that of miR-940 (Figure 5D). Moreover, we found that miR-940 was down-regulated in glioblastoma compared to non-tumor tissues according to an online dataset (GSE90605) (Figure 5E). To further confirm it, we analyzed the expression of miR-940 in 80 pairs of glioblastoma tissues and found that miR-940 was also down-regulated in tumor tissues (Figure 5F). Besides, we checked the correlation of lncHERG expression with miR-940 expression in glioblastoma. We found that the expression of lncHERG was negatively correlated with that of miR-940 in 80 glioblastoma samples (Figure 5G). In a word, lncHERG sponged miR-940 which was down-regulated in glioblastoma.

### LncHERG promoted cell proliferation, migration and invasion by sponging miR-940 in glioblastoma

**Figure 6:**
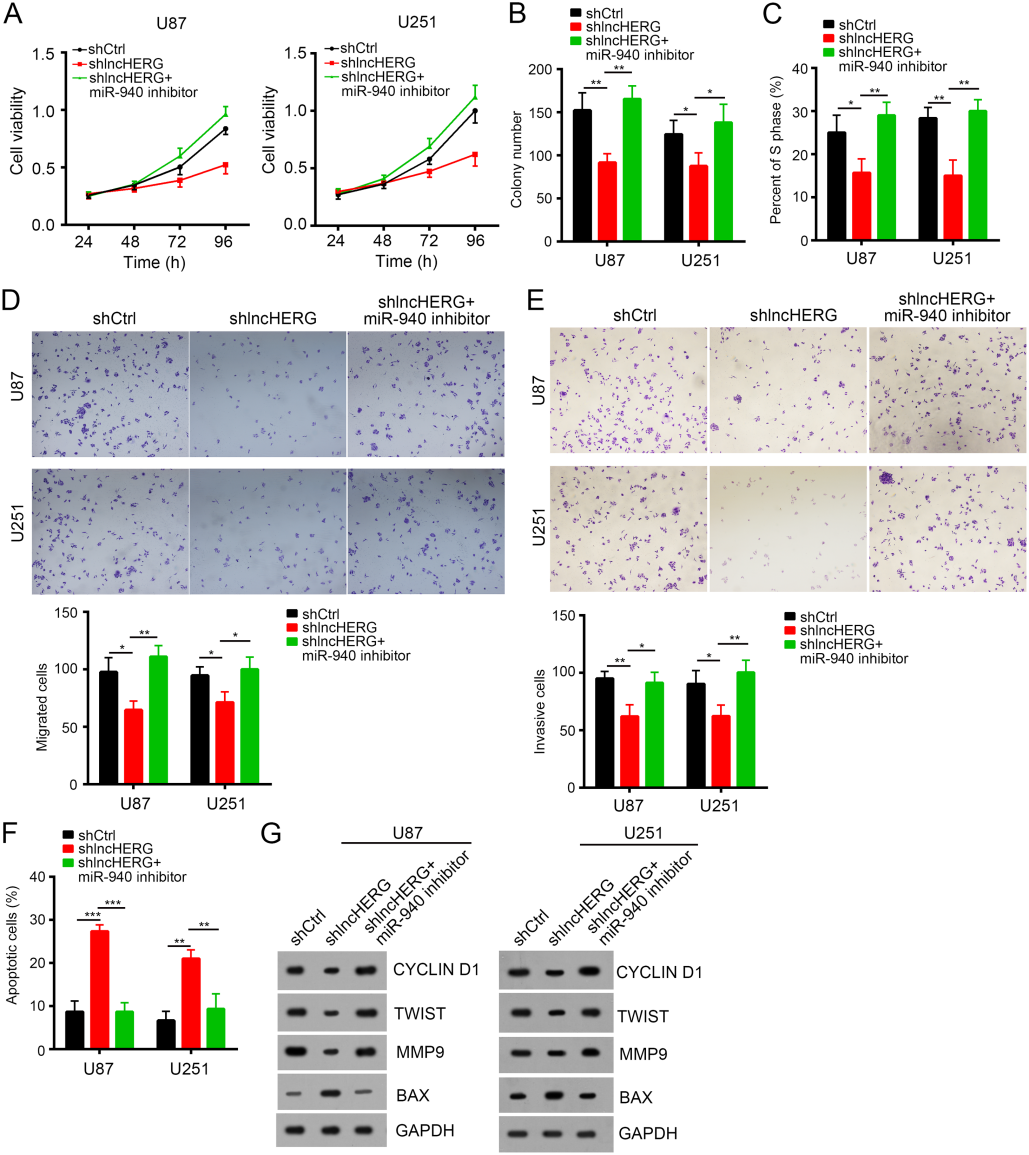
LncHERG promoted cell proliferation, migration and invasion by sponging miR-940 in glioblastoma **(A)** lncHERG knockdown inhibited cell proliferation while overexpression of miR-940 mimic in the meantime reversed this trend as shown by MTT assays. **(B)** lncHERG knockdown inhibited colony formation while overexpression of miR-940 mimic in the meantime promoted colony formation in U87 and U251 cells. **(C)** lncHERG silence inhibited cell entry into S phase while transfection with miR-940 mimic in the meantime promoted cell division. **(D)** and **(E)** lncHERG depletion remarkably inhibited cell migration and invasion while overexpressing miR-940 in the meantime promoted cell migration and invasion. **(F)** lncHERG knockdown promoted cell apoptosis while miR-940 overexpression in the meantime inhibited cell apoptosis. **(G)** lncHERG knockdown inhibited cell proliferation, migration and invasion while overexpressing miR-940 in the meantime reversed it in U87 and U251 cells. The protein levels of CYCLIN D1, TWIST, MMP9 and BAX were checked by Western blot. All data are representative of three independent experiments and expressed as mean ± SD. ^*^p<0.05, ^**^p<0.01 and ^***^p<0.001.

We have validated that lncHERG bond to miR-940 in glioblastoma. We wondered whether miR-940 was also a tumor suppressor and whether lncHERG inhibited tumor growth by sponging miR-940 in glioblastoma. To demonstrate it, we conducted MTT assays, we found that lncHERG knockdown inhibited cell proliferation while addition of miR-940 inhibitor reversed it in U87 and 251 cells (Figure 6A). Besides, lncHERG silence impaired colony formation while addition of miR-940 inhibitor in the meantime promoted the ability of colony formation (Figure 6B). Moreover, miR-940 inhibition promoted cell entry into cell cycle even under the condition of lncHERG knockdown (Figure 6C). We also checked the influence of miR-940 on migration and invasion. We found that transfection with miR-940 inhibitor promoted cell migration and invasion while lncHERG knockdown inhibited migration and invasion (Figure 6D and 6E). On the other hand, lncHERG depletion promoted cell apoptosis while miR-940 inhibition in the meantime decreased apoptosis (Figure 6F). Taken together, lncHERG promoted cell proliferation, migration and invasion by sponging miR-940 in glioblastoma (Figure 6G).

## DISCUSSION

Glioblastoma was the most common and aggressive brain cancer and gave rise to lots of deaths worldwide every year [1, 2]. However, the mechanism that regulates glioblastoma development and progression is largely unknown. As the development of bioinformatics, hundreds of lncRNAs have been identified in various tumors [27]. Many lncRNAs are involved in tumorgenesis and can regulate tumor growth and metastasis [16, 28]. In glioblastoma, some lncRNAs have been reported to be essential for tumor development. For example, lncRNA H19 promotes oncogenic effects in glioblastoma [29]. Long non-coding RNA taurine upregulated 1 promotes tumor-induced angiogenesis by inhibiting microRNA-299 in human glioblastoma [30]. lncRNA RP11-838N2.4 promotes the cytotoxic effects of temozolomide in glioblastoma cell lines [31]. However, there are still large amounts of lncRNAs whose function is unknown in glioblastoma. Therefore, in order to develop new and effective therapeutic management of GBM, it is an urgent and necessary to define the molecular mechanism that regulates the genesis of glioblastoma. In our study, we showed an uncharacteristic lncRNA lncHERG that was upregulated in glioblastoma than non-tumor tissues. We showed that lncHERG depletion impaired proliferation, migration and invasion of glioblastoma *in vitro* and *in vivo*. What's more, the higher expression of lncHERG in patients with glioblastoma indicated lower survival rate and poorer prognosis. Our study revealed the important role of lncHERG, which may serve as a new prognostic biomarker in glioblastoma.

LncRNAs may exert functions through various mechanisms including regulating chromatin remodeling and binding to miRNAs [32, 33]. To define the mechanism by which lncHERG exerted function in glioblastoma, we performed a target miRNA prediction. We found that there are 21 potential binding sites with miR-940 in lncHERG, which strongly indicated that lncHERG may sponge miR-940. Moreover, previous studies showed that miR-940 served as a tumor suppressor in various cancers including human nasopharyngeal carcinoma, prostate cancer, pancreatic ductal adenocarcinoma, hepatocellular carcinoma, ovarian cancer and triple-negative breast cancer [22, 24–26, 34, 35]. Nevertheless, the role of miR-940 in glioblastoma is totally unknown. To determine whether miR-940 is also a tumor suppressor in glioblastoma and whether lncHERG promoted tumor growth by inhibiting miR-940, we first conducted luciferase assays. We found that miR-940 mimic inhibited luciferase activity. Besides, we found that miR-940 overexpression decreased the mRNA levels of lncHERG and overexpressing lncHERG also inhibited the expression of miR-940. Moreover, the expression of lncHERG was negatively correlated with that of lncHERG in glioblastoma. All above indicated that lncHERG directly bond to miR-940 in glioblastoma. Then we found that miR-940 was down-regulated in glioblastoma compared to non-tumor tissues, which indicated that miR-940 may also act as a tumor suppressor. To validate it, we performed MTT, colony formation, migration and invasion assays. We found that lncHERG knockdown significant inhibited cell proliferation, migration and invasion, and promoted cell apoptosis. However, addition of miR-940 inhibitor in the meantime fully reversed these trends. Therefore, miR-940 acted as a tumor suppressor in glioblastoma and was a target of lncHERG. In different cancers, miR-940 seemed to target respective target. The target genes of miR-940 in glioblastoma remain to be explored further.

In summary, we screened out a unidentified lncRNA lncHERG that regulated tumor development and progression in glioblastoma by directly sponging miR-940. Our research revealed the pivot role of lncHERG/miR-940 axis in glioblastoma, which may provide a new sight on the treatment of glioblastoma.

## MATERIALS AND METHODS

### Patient samples

In this study, we obtained 80 pairs of glioblastoma samples from The Second Affiliated Hospital of Zhejiang University School of Medicine. We got consents of approving the usage of these samples in the study from all patients. All the experiments were approved by The Second Affiliated Hospital of Zhejiang University School of Medicine. The study protocol was approved by The Second Affiliated Hospital of Zhejiang University School of Medicine.

### Cell lines

Normal glial cell line (HA) and GBM cell lines (U87, U251 and LN229) were obtained from the Shanghai Cell Bank, Chinese Academy of Sciences (Shanghai, China). HA cells were cultured in Astrocyte Medium (AM). GBM cell lines were cultured in Dulbecco’s-modified Eagle medium (DMEM) F12 supplemented with 10% fetal bovine serum (FBS, Gibco) and 100×penicillin-streptomycin solution (Invitrogen Life Technologies).

### Construction and infection

Oligonucleotide encoding shRNA targeted lncHERG or a scramble shRNA was obtained from Sangon Biotech Inc. (Beijing, China) and constructed into pLKO.1-EGFP vector for lentivirus production. U87 and U251 cells were infected with lentivirus expressing shlncHERG or shCtrl. GFP^+^ stable cell lines were obtained by FACS isolation. shlncHERG sequence was as follows: 5'-GCCTTACAACAGCCTCTTTAC-3'.

### Cell proliferation assay

2×10^3^ cells/well were seeded in a 96-well plate and cultured at 37 °C for indicated times. Cells proliferation was detected by MTT assays. After cells were cultured for 24 h, 48 h, 72 h or 96 h, MTT was added to each group of wells and incubated for 1 h at 37 °C. Measurement was carried out in absorbance at 490 nm with a microplate reader according to the manufacturer’ s instruction.

### *In vitro* migration and invasion assays

For invasion assay, 1×10^5^ cells/well were seeded into the upper well of the Transwell chamber (BD Biosciences) coated with Matrigel (BD Biosciences) and cultured in serum-free DMEM. The lower chamber was filled with DMEM containing 10% FBS. Cells were incubated at 37°C for 48 h and then fixed with 4% paraformaldehyde and stained by crystal violet solution for 30 min. Cells on the top surface of the insert were removed with a cotton swab and counted under a microscope in five fields. Similar procedure was also performed for cell migration assay, except that the transwell membranes were not precoated with Matrigel.

### Reverse transcription and real-time PCR

Total RNAs from tumor samples were extracted using Trizol reagent (Life Technologies) according to the Manufacturer's instructions. The cDNA was synthesized from 5 μg of RNA using AMV reverse transcriptase (Fermentas, USA). Then real-time quantitative PCR was conducted using a standard SYBR Green PCR kit (Thermo Fisher Scientific, Rockford, IL, USA) on an ABI 7300 Real-Time PCR machine (Applied Biosystems, Foster City, CA, USA). The primer sequences were as follows: lncHERG forward: 5'-TTTTTGTGCCTGCCTCGTTG-3'; lncHERG reverse: 5'-GTTCGGCCTCAAGAAACTGC-3'; miR-940 forward: 5'-CCTGTCTTACTTTTCCGAAGGAC-3'; miR-940 reverse: 5'-TTGCTGTATTGTTGCCCATGT-3'.

### *In vivo* assay

6-week-old BALB/c nude mice were from HFK Biosciences. 4×10^6^ shCtrl or shlncHERG tumor cells were subcutaneously injected into nude mice. Tumor volumes were analyzed at indicative time points. And the tumor weights were measured 7 weeks after injection. Animal experiments were performed in accordance with relevant guidelines and regulations of the Institutional Animal Care and Use Committees at The Second Affiliated Hospital of Zhejiang University School of Medicine, and protocols were approved by the Institutional Animal Care and Use Committees at The Second Affiliated Hospital of Zhejiang University School of Medicine.

### Luciferase reporter assay

293T cells were seeded into a 24-well plate. For lncHERG and miR-940 interaction, cells were co-transfected with wild-type, mutated lncHERG reporter plasmid or pRL-TK vector, and miR-940 mimics or miR-940 inhibitor. Luciferase assays were conducted 24 h after transfection using the Dual Luciferase Reporter Assay System (Promega, WI, USA).

### *In situ* hybridization

LncHERG expression in GBM tissues were analyzed using biotin-labeled specific lncHERG probes. Paraffinized sections were deparaffinized with xylene and 100% ethanol. Then sections were incubated with biotin-labeled probes for 18 h at 40 °C. DAB substrate was used for colorimetric detection of lncHERG. Finally, the sections were co-stained with hematoxylin, followed by dehydration in graded alcohols and xylene. lncHERG probe sequences as follows: #1: 5'-GGCAGGCACAAAAATGGTCT-3'; #2: 5'-AGGAACGTGGTCTGGAAGGC-3'.

### Northern blot

RNAs were extracted from GBM samples using TRIZOL (Invitrogen). LncHERG and GAPHD probes for Northern blot were achieved by Biotin RNA labeling mix (Roche). The RNA samples were separated by electrophoresis and transferred to NC membrane. Then the membranes were incubated with hydration buffer containing probes. Finally, RNA signal was detected with Chemiluminescent Nucleic Acid Detection Module (Thermo Scientific).

### Statistical analysis

All statistical analyses were performed using SPSS 20.0 (IBM, SPSS, Chicago, IL, USA) and GraphPad Prism. Student's t-test and one-way ANOVA were used to analyze 2 or multiple groups, respectively, for statistical significance. Pearson correlation coefficient analysis was used to determine the correlations. The overall survival curves were calculated with the Kaplan-Meier method and were analyzed with the log-rank test. P< 0.05 was considered statistically significant in all cases.
